# Computed Tomography Features and Diagnostic Pitfalls of Bronchial Chili Pepper Aspiration: Distinctive Morphologies and the False-Negative Peel—A Radiologic–Endoscopic Report of Two Cases

**DOI:** 10.3390/jcm15218239

**Published:** 2026-10-23

**Authors:** Ying Li, Rui Yin, Boxin Li, Hong Zhang

**Affiliations:** Department of Radiology, Tianjin Chest Hospital, Tianjin University, Tianjin 300222, China; liying_tmu@163.com (Y.L.);

**Keywords:** foreign body aspiration, chili pepper, computed tomography, bronchoscopy, case report

## Abstract

**Background**: Tracheobronchial foreign body aspiration (FBA) is a recognized clinical emergency; however, aspiration of chili fragments represents an underdiagnosed subtype, particularly in older adults. This report aims to characterize the spectrum of computed tomography (CT) manifestations and diagnostic pitfalls associated with chili fragment aspiration. **Case Presentation**: We report two elderly patients who presented with subacute respiratory symptoms initially mimicking post-obstructive pneumonia or bronchogenic malignancy. Both underwent thin-slice thoracic CT and flexible bronchoscopy. Case 1: A 65-year-old female whose CT revealed a sharp, V-shaped hyperdense opacity with a mean attenuation value of +598 HU, within the right lower lobe bronchus associated with distal obstructive atelectasis. Flexible bronchoscopy confirmed and extracted a chili fragment. Case 2: A 63-year-old male demonstrated a well-circumscribed, punctate hyperattenuating focus (+173 HU) in the right lower lobe bronchus, corresponding to an aspirated chili seed. Notably, an accompanying chili peel was retrieved endoscopically but was not separately identifiable on retrospective CT review. Both patients achieved complete symptom resolution following endoscopic extraction. **Conclusions**: In cases of unexplained pneumonia, particularly in elderly individuals and those with relevant dietary habits, the possibility of chili fragment aspiration should be considered. CT imaging of hyperdense, V-shaped, or punctate opacities within the bronchial tree may provide valuable diagnostic clues. However, chili peels may not be identifiable on retrospective CT review. Radiologists should be aware that a negative chest CT does not exclude the presence of an organic foreign body. Maintaining clinical vigilance and integrating endoscopic evaluation are essential for accurate diagnosis and complete removal of the foreign body.

## 1. Introduction

Tracheobronchial foreign body aspiration (FBA) represents a significant diagnostic and therapeutic challenge in both pediatric and older adult populations [1,2]. In contrast to the classic, dramatic presentation of acute choking in pediatric cases, older patients frequently present with insidious, subacute respiratory symptoms such as unresolving cough, localized wheezing, and recurrent obstructive pneumonia [3,4]. The etiology of aspirated organic matter is largely shaped by regional dietary practices [5,6]. While tracheobronchial aspiration of chili peppers is less frequently documented, the high capsaicin content and potent chemical irritancy of chili peppers provoke profound mucosal inflammation, leading to florid mucosal edema, exuberant granulation tissue, and purulent exudates [7]. These reactive changes often mimic chronic airway diseases or central bronchogenic malignancies, contributing to substantial diagnostic delays [8].

Computed tomography (CT) serves as the first-line imaging modality for suspected FBA. On thoracic CT—particularly in mediastinal window settings—these botanical foreign bodies can manifest as hyperattenuating endobronchial structures with distinct geometric morphologies, including V-shaped, U-shaped, or curvilinear configurations [9]. Although these radiological findings lack absolute diagnostic specificity, they can critically narrow the differential diagnosis. Nonetheless, distal migration of chili fragments and associated inflammatory reactions—such as granulation tissue formation, mucosal edema, and purulent secretions—can obscure these foreign bodies [4,8]. Bronchoscopy remains both the diagnostic gold standard and the definitive therapeutic intervention.

Herein, we report two elderly patients with bronchial chili pepper aspiration to describe key imaging features and explore potential diagnostic pitfalls.

## 2. Case Presentations

### 2.1. Case 1

A 65-year-old female presented to our pulmonology department with a 2-week history of persistent, non-productive cough and low-grade fever, with body temperatures fluctuating between 37.5 °C and 38.5 °C. Empirical treatment with oral antibiotics at a local clinic had failed to confer clinical improvement. She had no documented history of acute choking, neurological deficits, or dysphagia. Physical examination demonstrated reduced breath sounds over the right lower lobe; no wheezes, rales, or rhonchi were auscultated. Laboratory evaluation demonstrated mild leukocytosis (white blood cell count: 10.14 × 10^9^/L).

Non-contrast thoracic CT examinations were performed on a 256-slice Revolution CT scanner (GE Healthcare, Milwaukee, WI, USA). Acquisition parameters were: tube voltage, 140 kVp; tube current-exposure time, 227 mAs. Reconstruction parameters included a slice thickness of 1.25 mm with an interval of 1.25 mm, using a lung kernel and standard kernel, combined with adaptive statistical iterative reconstruction-V (ASiR-V) at 40%.

**Figure 1:**
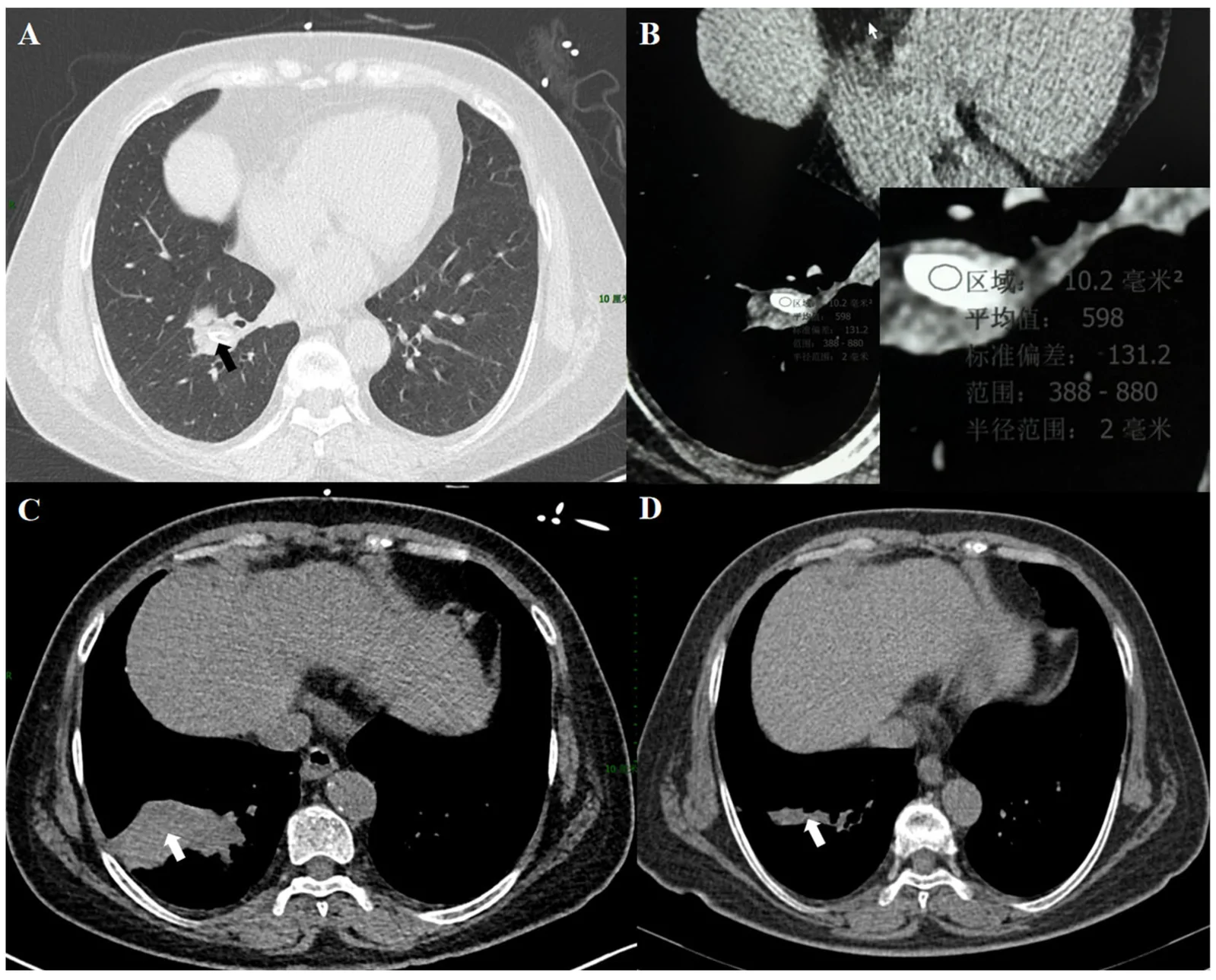
Window settings: lung window, width 1500 HU; level −600 HU. Mediastinal window, width 400 HU; level 40 HU. (**A**) Axial CT on lung window showing a sharp, V-shaped hyperdense foreign body lodged (black arrow) in the right lower lobe bronchus. Green Chinese characters in (**A**,**C**) indicate 10 cm. (**B**) Magnified mediastinal window view with ROI cursor measuring the CT attenuation of the chili fragment (mean: +598 HU). Area denotes the cross-sectional area of the ROI (10.2 mm^2^); Standard deviation (SD) reflects the dispersion of pixel values around the mean (131.2); Range gives the minimum and maximum pixel values (388 HU–880 HU); and Radius specifies the radius of the circular ROI (2 mm). (**C**) Axial CT on mediastinal window showing distal obstructive atelectasis and peribronchial consolidation (white arrow). (**D**) Axial CT on mediastinal window conducted 50 days after the procedure demonstrates a significant decrease in pulmonary lesions relative to the previous examination (white arrow).

Thoracic CT revealed a well-defined, V-shaped hyperdense structure lodged within the lumen of the right lower lobe bronchus on lung window settings (window width: 1500 HU; window level: −600 HU) (Figure 1A, black arrow). On mediastinal window settings (window width: 400 HU, window level: 40 HU), region-of-interest (ROI) measurements (ROI size: 10.2 mm^2^) demonstrated a mean attenuation value of +598 HU (range: +388 to +880 HU) (Figure 1B). The ROI was placed within the central portion of the V-shaped hyperdense structure on the axial slice showing its largest cross-section, avoiding the bronchial wall and adjacent air. This attenuation value corresponded to the chili pericarp, as confirmed by examination of the retrieved specimen. Distal to the endobronchial lesion, subsegmental atelectasis and patchy consolidation were observed on the mediastinal window (Figure 1C, white arrow).

**Figure 2:**
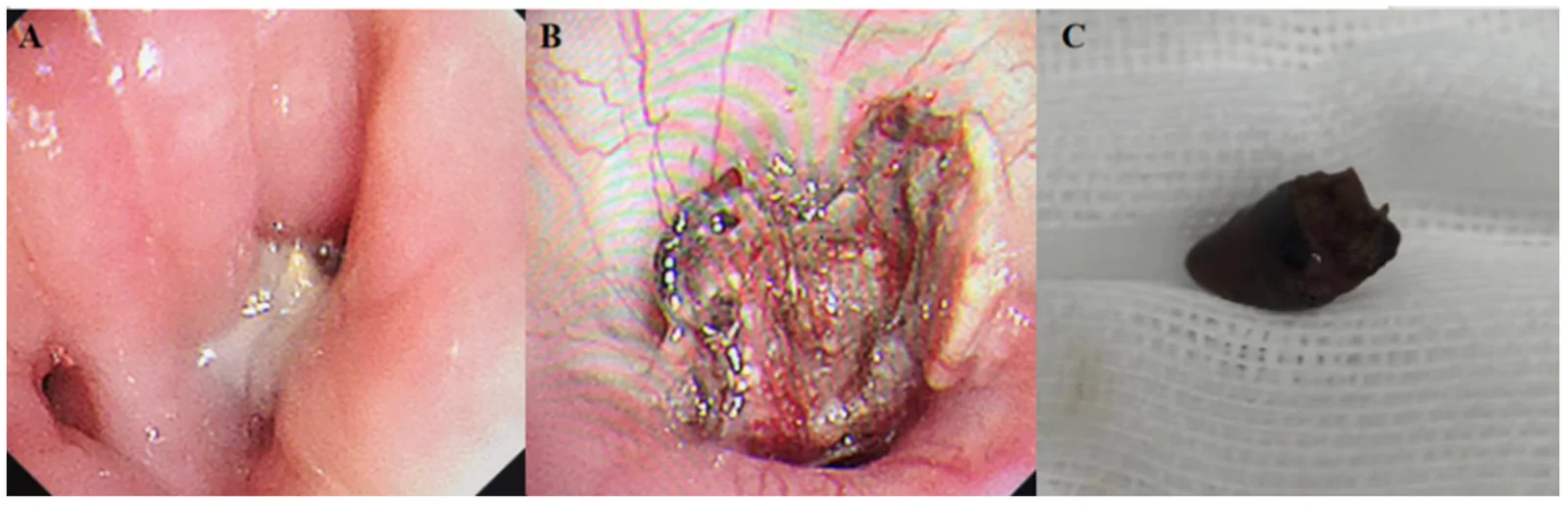
(**A**,**B**) Bronchoscopic view demonstrating the folded chili fragment wedged in the bronchus with surrounding granulation. (**C**) Gross photographic specimen of the retrieved chili fragment.

Given the high suspicion of an endobronchial foreign body or calcified endobronchial lesion, flexible bronchoscopy was performed under local anesthesia. Endoscopic visualization revealed a firm, folded organic foreign body wedged at the bifurcation of the right lower basal bronchus, surrounded by moderate mucosal hyperemia and granulation tissue (Figure 2A,B). The object was successfully extracted in one piece using endoscopic forceps. Gross examination confirmed a rigid chili pericarp fragment with no associated seeds (Figure 2C). Post-procedure CT at 50 days on mediastinal window showed partial resolution of the distal atelectasis with improved clinical symptoms and uneventful recovery (Figure 1D, white arrow).

### 2.2. Case 2

A 63-year-old male presented with a 3-month history of persistent coughing. The patient did not report significant sputum production, and the cough exhibited no regular temporal pattern. No other symptoms were noted. Despite symptomatic treatment at multiple hospitals, the patient showed no clinical improvement. Due to disease exacerbation, the patient was hospitalized for diagnosis and treatment. Upon focused re-interviewing, he recalled an episode of severe coughing while consuming a spicy meal approximately 3 months prior.

CT examination was conducted under the identical imaging protocol and equipment described in Case 1.

**Figure 3:**
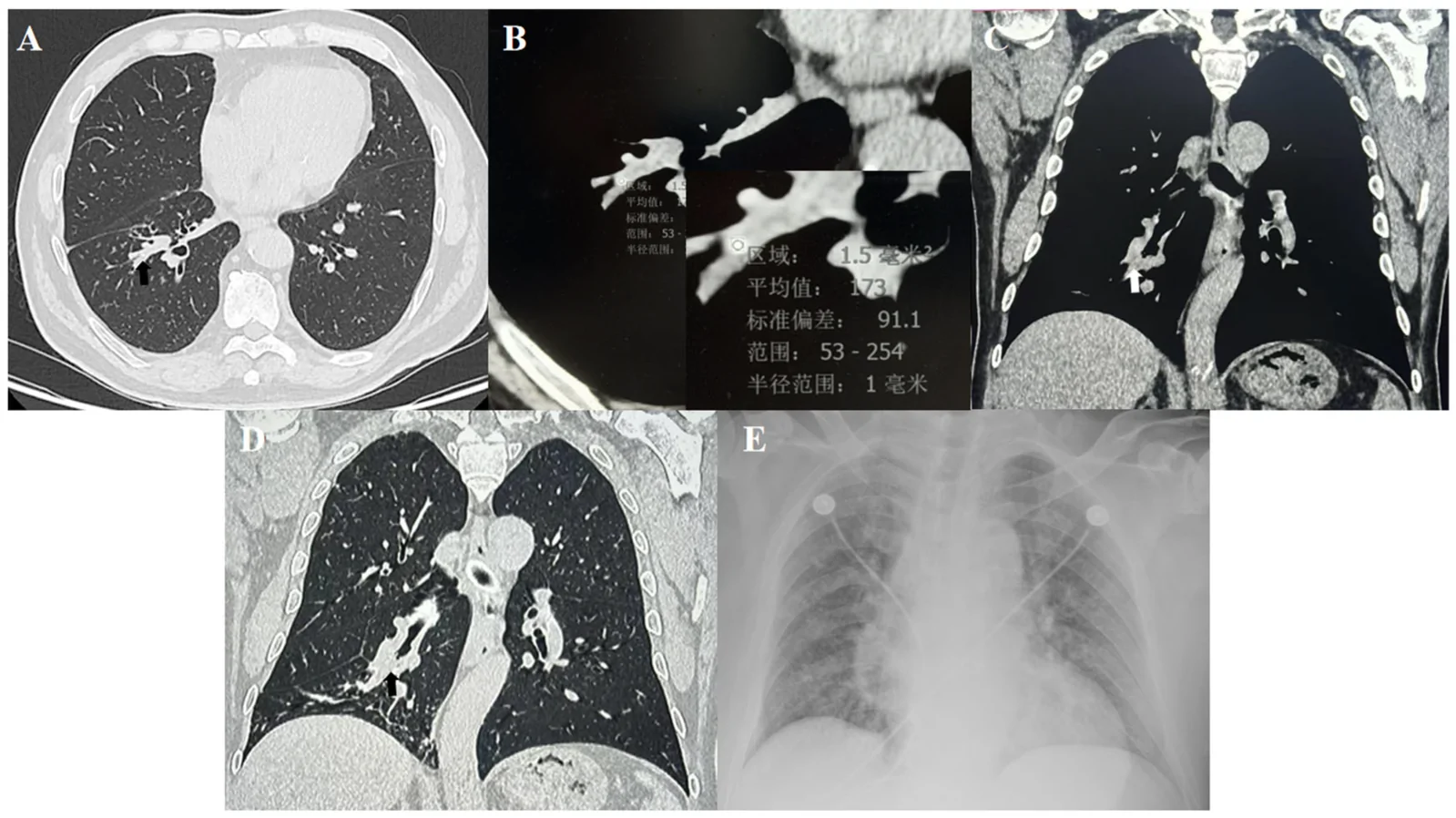
Window settings: lung window, width 1500 HU; level −600 HU. Mediastinal window, width 400 HU; level 40 HU. (**A**) Axial CT on lung window displaying a well-circumscribed punctate hyperdense opacity (black arrow) within the right lower basal bronchus. (**B**) Magnified mediastinal window view with ROI cursor measuring the CT attenuation of the seed (+173 HU). Area denotes the cross-sectional area of the ROI (1.5 mm^2^); Standard deviation (SD) reflects the dispersion of pixel values around the mean (173); Range gives the minimum and maximum pixel values (53 HU–254 HU); and Radius specifies the radius of the circular ROI (1 mm). (**C**) Retrospective multi-planar review on the mediastinal window of pre-procedural thin-slice CT scans: no hyperattenuating opacity was identified at the site of the chili peel (white arrow). (**D**) Retrospective multi-planar review on the lung window of pre-procedural thin-slice CT scans: no hyperattenuating opacity was identified at the site of the chili peel (black arrow). (**E**) Follow-up Digital Radiography (DR) on postoperative day 3 demonstrated partial resolution of the distal atelectasis and improvement in clinical symptoms.

Thoracic CT revealed a solitary, hyperdense punctate focus lodged within the right lower lobe basal bronchus on lung window settings (window width: 1500 HU; window level: −600 HU) (Figure 3A, black arrow). Attenuation measurement on the mediastinal window (window width: 400 HU, window level: 40 HU), ROI (size: 1.5 mm^2^) showed a mean attenuation value of +173 HU (range: +53 to +254 HU) (Figure 3B). Because of the small size of the focus, the ROI was placed over its center on the axial slice with the greatest visible attenuation; the ROI was fully confined within the lesion margins. This attenuation value corresponded to the chili seed, as confirmed by examination of the retrieved specimen, rather than to the concurrently retrieved chili peel. Distal to the endobronchial lesion, subsegmental atelectasis and patchy consolidation were observed.

**Figure 4:**
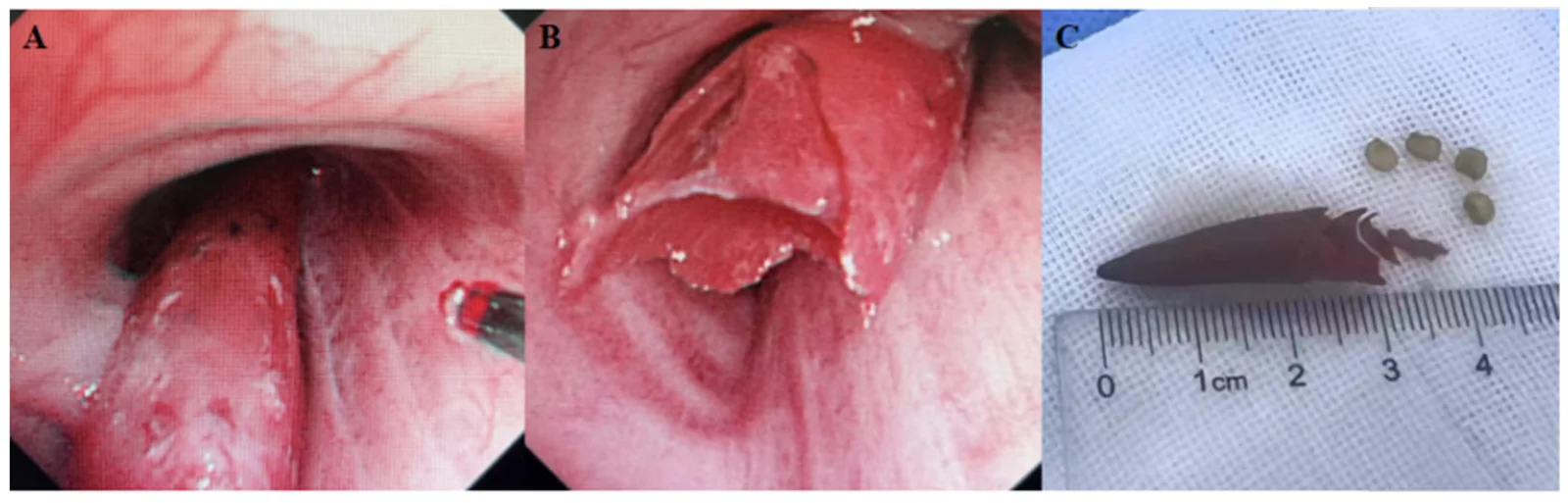
(**A**,**B**) Bronchoscopic image showing a chili fragment lodged at the orifice of the right lower lobe bronchus. (**C**) Gross photographic specimen of the retrieved chili fragment.

A flexible bronchoscopy was performed for definitive evaluation and foreign-body removal. Endoscopic examination revealed an object within the orifice of the right lower lobe bronchus (Figure 4A,B). The foreign body was retrieved in multiple steps using biopsy forceps, a CO_2_ cryoprobe, and a retrieval basket. Both chili pepper peel and seeds were recovered. The seed appeared yellow and round, consistent with a seed-like morphology and corresponding to the punctate hyperdense focus seen on CT. The retrieved pepper peel measured approximately 35 mm in length (Figure 4C). Notably, a retrospective, multi-planar review of the pre-procedural thin-slice CT scans—including multiplanar reformations (MPR) in axial, coronal, and sagittal planes on both lung and mediastinal windows—failed to identify any hyperattenuating opacity at the site of the chili peel (Figure 3C,D, white/black arrow). Follow-up Digital Radiography (DR) on postoperative day 3 demonstrated partial resolution of the distal atelectasis and improvement in clinical symptoms (Figure 3E).

The form in which the chili was ingested (dried, fresh, or cooked in oil) was not documented and could not be established from the patient’s history.

## 3. Discussion

We report two elderly patients with aspiration of chili peppers, a population particularly susceptible to bronchial foreign-body aspiration. In both cases, the foreign bodies impacted within the right lower lobe bronchus, conforming to the classic anatomical predisposition dictated by the wider caliber and steeper vertical trajectory of the right mainstem bronchus. Notably, the CT findings differed between the two patients: the first demonstrated the typical hyperattenuating “V-shaped” morphology described in the previous literature, whereas the second revealed a rare punctate hyperdensity, in contrast to the ring-shaped appearance more often reported [9]. Furthermore, in the second patient, while the primary presumed seed was detected on initial CT, an associated 35 mm chili peel retrieved during bronchoscopy was not separately identifiable on retrospective CT review. This case highlights a failure to detect an additional component within a composite foreign body, offering valuable educational insight into the imaging spectrum of this condition.

FBA is infrequently detected in adults. In a single-centre study, 32 of 12,588 adult flexible bronchoscopies performed over 25 years were found to have an airway foreign body, corresponding to a detection rate of only 0.25% [10]. Previous studies have reported that foreign bodies in adult patients were more frequently located in the right bronchial tree, especially in the bronchus of the right lower lobe [11,12]. The larger diameter, more vertical orientation, and greater airflow of the right mainstem bronchus may contribute to the higher frequency of aspiration into the right bronchial tree [13]. This is consistent with the occurrence site of the cases we provided.

Chili aspiration accounts for only a small proportion of all airway foreign body cases, yet it represents a distinct subtype characterized by specific clinical and inflammatory features [2,14,15]. Chemically, chili peppers contain high concentrations of capsaicin (8-methyl-N-vanillyl-6-nonenamide), a potent agonist of transient receptor potential vanilloid 1 (TRPV1) receptors expressed on airway sensory nerves. Capsaicin induces local vasodilation and bronchospasm, and accelerates mucosal granulation tissue proliferation [7]. Clinically, patients often present with paroxysmal cough (productive or non-productive), which may be accompanied by chest pain, dyspnea, or symptoms of obstructive pneumonia (e.g., fever, purulent sputum) [14,16]. These symptoms overlap with those of common respiratory diseases, often leading to misdiagnosis and delayed intervention [4].

Radiologically, the internal architecture and composition of aspirated chili peppers dictate their CT appearance. Dense, lipid-rich seeds typically manifest as well-circumscribed, punctate, or ovoid hyperattenuating foci, whereas dried pericarp fragments characteristically display sharp, folded, V-shaped morphologies with hyperattenuating margins clearly delineated on mediastinal window settings [9]. This direct sign is best appreciated on mediastinal window settings and is suggestive—but not specific—for aspirated chili fragments. Other indirect CT findings may include localized bronchial wall thickening, luminal narrowing, peripheral consolidation, post-obstructive atelectasis, ground-glass opacities, bronchiectasis, and even fibrotic streaking distal to the site of obstruction [8].

Dried, fresh, and oil-cooked chilies vary in water content, lipid content, and tissue compactness, which may influence their baseline CT attenuation. Furthermore, in Chinese culinary practices, stir-frying can reduce capsaicinoid content by approximately 39% to 53% through thermal degradation and lipid dissolution, rendering cooked chili considerably less pungent than its raw counterpart [17]. This heat-induced reduction in capsaicin bioavailability may attenuate the severity of transient receptor potential vanilloid 1 (TRPV1)–mediated airway inflammatory responses compared with the aspiration of raw chili fragments. However, the exact form in which the chili was ingested was not documented in either of our cases, precluding an assessment of its specific contribution.

In addition, the interval between aspiration and CT examination differed substantially between the two patients (approximately 2 weeks in Case 1 versus 3 months in Case 2). Desiccated vegetative matter is inherently hygroscopic and well known to swell, soften, and provoke localized inflammation within the tracheobronchial tree [18]. Prolonged lodgement in a moist, infected airway could facilitate progressive hydration, structural softening, and gradual degradation of the pericarp, potentially shifting its CT attenuation toward that of surrounding endobronchial secretions. Because no previous studies have serially measured the attenuation of aspirated chili pericarp over time, this mechanism remains hypothetical. Moreover, potential confounding factors—including fragment size, scanner protocols, and encasement by inspissated mucus or granulation tissue—obscure the isolated effect of lodgement duration.

In Case 1, a V-shaped hyperdense structure (+598 HU) corresponded to the chili pericarp. At bronchoscopy, the fragment was found to be rigid and folded. We speculate that this folded morphology may have increased the effective thickness of attenuating material along the X-ray beam path relative to a single-layer peel, possibly accounting in part for its higher density and visibility. In Case 2, a punctate focus (+173 HU) was attributed to the seed; because of its small size, it was likely affected by partial-volume averaging with surrounding air or soft tissue, such that the measured +173 HU may underestimate its true attenuation [19]. In addition, kernel choice and reconstruction algorithm can affect image noise, edge sharpness, and measured CT numbers, particularly in small structures [20,21,22]. By contrast, the approximately 35 mm peel in Case 2 was not separately identifiable on CT, even upon retrospective review of thin-section multiplanar reformations. This may be because a thin, unfolded peel approaches or falls below the limits of spatial resolution achievable with this imaging protocol, given the combined effects of slice thickness, detector sampling, and reconstruction parameters [19,20]. In severe cases of partial-volume averaging, a thin structure’s attenuation may be averaged so completely with adjacent air or secretions that it becomes radiographically occult [19]. Furthermore, adjacent granulation tissue and retained secretions may have obscured the margins of this low-contrast fragment and contributed to averaging at the lesion edge, as soft-tissue-density material abutting a thin fragment can reduce its conspicuity [14,23].

The educational value of these two cases lies precisely in their differing CT appearances, which underscore that chili aspiration does not adhere to a single, uniform radiological sign. Radiologists should be familiar with the CT features of aspirated chili fragments, which typically appear as intraluminal, well-circumscribed, radiopaque hyperdensities with varying morphology. Although these features are not specific to this condition, heightened awareness may aid in prompt diagnosis. Crucially, despite evident bronchial impaction, thin chili pepper membranes may not be reliably identified on thoracic CT. These cases convey two important clinical lessons. First, focused history-taking remains paramount: an elicited history of choking during a meal should immediately prompt suspicion of aspiration, even when CT manifestations are subtle or atypical. Second, these findings highlight the necessity of complete and meticulous bronchoscopic inspection. Bronchoscopists must not terminate the examination immediately after retrieving a solitary foreign body (such as a seed); instead, they must systematically inspect the tracheobronchial tree to exclude concomitant, radiologically occult components, such as pericarp fragments. As long as clinical suspicion persists, flexible bronchoscopy must remain the definitive diagnostic and therapeutic standard, irrespective of negative or equivocal CT signs [5].

This study has several limitations. First, the small sample size (only two cases) inherently limits generalizability and precludes quantitative assessment of the diagnostic sensitivity or specificity of the described CT features, thereby restricting external applicability. Second, the lack of standardized macroscopic photography with a calibrated measurement scale for the specimen in Case 1 and the solitary seed in Case 2 precluded exact ex vivo metric documentation. Third, the retrospective, single-center design may introduce selection bias, as only patients requiring intervention for persistent symptoms were included, potentially excluding mild cases that resolved spontaneously and biasing the results toward a more severe spectrum. Fourth, there are currently no validated radiologic diagnostic criteria specific to chili fragment aspiration; the observed CT findings (e.g., V-shaped or nodular hyperattenuating structures) are not pathognomonic and may overlap with other organic or inorganic radiodense foreign bodies. Finally, the definitive radiological non-visualization of the accompanying chili peels remains a diagnostic hurdle, largely attributable to partial-volume averaging effects, minimal physical thickness, motion artifacts, and obscuration by surrounding inflammatory exudates or granulation tissue.

## 4. Conclusions

In conclusion, this case series delineates the radiological spectrum of tracheobronchial chili pepper aspiration, underscoring its morphological heterogeneity on thoracic computed tomography. Endobronchial hyperattenuating structures manifesting as distinct “V-shaped” or punctate morphologies should heighten clinical suspicion for botanical foreign bodies. However, these features lack absolute specificity, and associated components—particularly thin pericarp fragments—may remain radiographically occult, precipitating false-negative evaluations. In older adults presenting with unresolved focal pulmonary infiltrates, recurrent atelectasis, or lobar consolidation (predominantly involving the right lower lobe), the absence of a discernible foreign body on CT does not preclude organic foreign-body aspiration, especially when coupled with a collateral history of choking during meals. Clinicians and radiologists must maintain a high index of suspicion and pursue prompt flexible bronchoscopy to establish a definitive diagnosis and avert irreversible airway complications.

## Data Availability

The data supporting this case report are available from the corresponding author on reasonable request, subject to patient privacy and institutional regulations.

## Abbreviations

The following abbreviations are used in this manuscript:

CT	Computed tomography
FBA	Foreign body aspiration
HU	Hounsfield Units
DR	Digital Radiography
